# Ethical considerations for research involving pregnant women living with HIV and their young children: a systematic review of the empiric literature and discussion

**DOI:** 10.1186/s12910-021-00601-x

**Published:** 2021-04-01

**Authors:** Catherine G. Raciti, Leslie A. Enane, Katherine R. MacDonald, Elizabeth C. Whipple, Mary A. Ott, Megan S. McHenry

**Affiliations:** 1 Indiana University-Purdue University – Indianapolis, Indianapolis, IN USA; 2 Department of Pediatrics, The Ryan White Center for Pediatric Infectious Disease and Global Health, Indiana University School of Medicine, 705 Riley Hospital Drive, Room 5853, Indianapolis, IN 46202 USA; 3 Department of Adolescent and Young Adult Medicine, Children’s National Hospital, Washington, DC USA; 4 Division of Adolescent Medicine, Department of Pediatrics, Indiana University School of Medicine, Indianapolis, IN USA; 5 Indiana University School of Medicine, Ruth Lilly Medical Library, Indianapolis, IN USA

**Keywords:** Pregnant women, HIV, Research, Ethics, Belmont Report

## Abstract

**Background:**

The proper and ethical inclusion of PWLHIV and their young children in research is paramount to ensure valid evidence is generated to optimize treatment and care. Little empirical data exists to inform ethical considerations deemed most critical to these populations. Our study aimed to systematically review the empiric literature regarding ethical considerations for research participation of PWLHIV and their young children.

**Methods:**

We conducted this systematic review in partnership with a medical librarian. A search strategy was designed and performed within the following electronic databases: Ovid MEDLINE, Embase and CINAHL. We screened titles and abstracts using the following inclusion criteria: (1) a study population of PWLHIV or children under 5 years of age; and (2) collection of qualitative or quantitative data regarding ethics of research participation. Excluded were reviews, commentaries, policy statements, clinical care-related ethics concerns, abstracts, case studies, or studies unrelated to HIV research. Studies were appraised for quality, data were extracted, and studies were qualitatively analyzed using a principle-based ethical framework within the Belmont Report.

**Results:**

Of the 7470 titles identified, 538 full-text articles were reviewed for eligibility and only three articles met full criteria for inclusion within this review. While we allowed for inclusion of studies involving young children born to mothers with HIV, only articles focused on PWLHIV were identified. Within the results of these studies, four themes emerged: (1) adequacy of informed consent; (2) consideration of paternal involvement; (3) balancing risks; and (4) access to research and treatment. A strength of this review is that it included perspectives of international research investigators, community leaders, and male partners. However, only two studies collected empiric data from PWLHIV regarding their experiences participating in research

**Conclusion:**

Researchers and funding agencies should be aware of these considerations and appreciate the value of and critical need for formative research to ensure clinical trials involving PWLHIV promote ethical, well-informed research participation and, ultimately, improve care outcomes. More research is needed to create a comprehensive ethical framework for researchers when conducting studies with PWLHIV.

## Background

Over the last three decades, tremendous progress has been made in HIV prevention and care. HIV infection—once a devastating death sentence—is now a manageable chronic disease with appropriate antiretroviral treatment, and preventable through pre-exposure prophylaxis, treatment-as-prevention, and prevention of mother-to-child transmission. Perinatal HIV, however, continues to be a critical target for further intervention. Advancements in HIV care, impacting nearly 38 million individuals living with HIV today, are informed by innovative and rigorous global research [1]. Yet, there are populations, including pregnant women and young children, who have limited access to HIV research. Barriers include ethical concerns such as stigma, racism, and discrimination based upon gender or sexual minority status, cultural differences related to informed consent, health literacy, and the regulatory burden for research with populations perceived as being vulnerable. The proper and ethical inclusion of PWLHIV and their young children in research is paramount to ensure valid evidence is generated to optimize treatments and care [2].

Ethical frameworks, such as the CIOMS guidelines, identify women living with HIV, pregnant women, and young children as populations that should be presumed eligible for research and provide guidance on considerations to undertake when including them [3]. The CIOMS guidelines emphasize the importance of establishing regulated and ethical processes when designing research studies, considering the unique physiology of pregnant women, conducting a risk assessment, and in obtaining informed consent [3]. Despite the urgency to promote research relevant to pregnant women’s health needs, historical events and different ideologies prevent researchers from including this population in research [4]. And while the CIOMS guidelines encourage the inclusion of pregnant women, using these guideline alone, researchers and academic institutions still feel hesitant or ill-equipped to address ethical considerations for research involving these populations [5]. This results in severe disparities regarding access to critical research for PWLHIV [6].

Narrative reviews or expert opinions outlining philosophical concerns related to ethical research standards often group PWLHIV and their children with all potential research participants or lack empiric data supporting best practices for these individuals [7, 8]. As pregnancy status, gender, and age may affect HIV-related outcomes, the care needed for PWLHIV should not be generalized with non-pregnant women and men. However, without increased guidance, researchers will continue to overprotect PWLHIV by excluding them from studies, resulting in under protection when it comes to their clinical care [4]. Therefore, more specific data from the field are needed to inform ethical considerations deemed most critical to the targeted populations. Our study aimed to systematically review the empiric literature regarding ethical complexities and considerations for research participation of PWLHIV and their young children.

## Methods

### Search strategy

We conducted a systematic search using a protocol designed together with a medical librarian (EW) in accordance with PRISMA guidelines [9]. Ovid MEDLINE, Embase and CINAHL were searched on November 26, 2019 using a comprehensive search strategy. We then searched Google Scholar, Cochrane Database for Systematic Reviews, and the bibliographies of pertinent articles. Keywords included terms for “HIV,” “Ethics,” and “Research Participation.” The full search strategy from Ovid MEDLINE is provided in “Appendix 1”.

The initial screening of titles and abstracts was performed by three independent reviewers (MSM; KM; LAE) The process was standardized by using a written protocol and a set of a priori codes based on the inclusion criteria. Articles were excluded if they did not report qualitative or quantitative data gathered during a research study regarding ethical issues in HIV research. Full texts of the remaining articles were independently reviewed (MSM and CGR) to determine whether articles met the complete predetermined eligibility criteria, with disagreements between the reviewers settled through discussion and consensus. MO was available as a third reviewer if consensus was not met, although this was not utilized within this review.

### Eligibility

Included in this review were studies or articles involving: (1) a study population of PWLHIV or children under 5 years of age; and (2) collection of qualitative or quantitative data regarding ethics or social complexities influencing participation in research. Excluded were: studies primarily focused on ethics in the clinical care of patients with HIV; studies regarding ethical issues around women becoming pregnant while participating in research; review, editorial, commentary, or opinion articles; organizational policy statements; and published abstracts without full-text publications. We additionally limited articles to those published after 1985, those involving human subjects, and those in the English language.

Articles focusing on ethical issues specific to non-pregnant adolescents living with HIV were included in a separate systematic review [10].

### Ethical framework

The principle-based ethical framework within the Belmont Report is a cornerstone of ethical frameworks both national and internationally, including the CIOMS guidelines [11]. As such, this principle-based ethical framework was used in this review to evaluate themes arising from the included article. The three primary ethical principles outlined were Beneficence, Respect for Persons, and Justice.

### Data extraction

Study data were extracted into an electronic table by one reviewer (CGR) and cross-checked independently by the second reviewer (MSM), including study design, study population, study settings, ethical issues explored, limitations, and quality assessment (Appendix 2). Data were described qualitatively and mapped within the principle-based framework when appropriate. To identify overall ethical themes across studies, there was a thematic synthesis conducted, consisting of three stages [12]. Two reviewers (MSM & CGR) independently coded all articles that met study inclusion criteria. Overall descriptive themes were developed during the second stage. Each of which was analyzed through the three main principles outlined in the Belmont Report, generating four analytical themes in the final stage of the thematic synthesis [12].

## Results

**Fig. 1:**
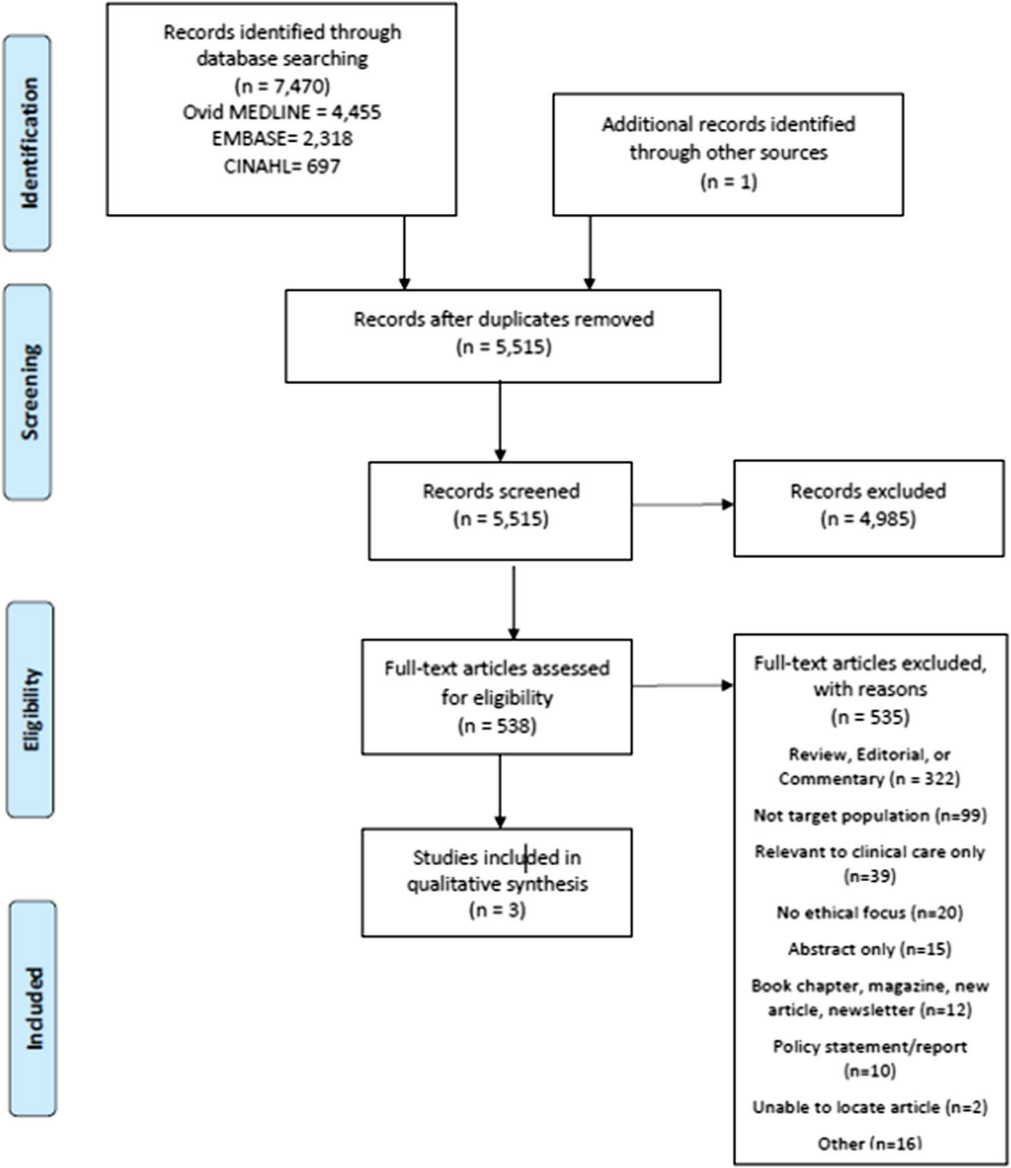
PRISMA flow diagram of the study selection process

The initial search resulted in 7470 articles. After duplicates were removed and one additional article was identified outside of our search, 5515 articles were screened, resulting in 538 full-text articles reviewed for eligibility (Fig. 1). Of the 538 articles screened, 535 articles were excluded from the review. The majority were excluded on account of being a review/editorial, missing target population, or only pertaining to ethics in clinical care. Only three articles remained for inclusion within the systematic review (Table 1), and all were primarily focused on pregnant women.

**Table 1:** Study characteristics

References	Country	Study design	Sample size	Relevant data collection methods	Key results	Notes	Themes	Quality Assessment using STROBE guidelines
Corneli et al. [13]	Malawi	Qualitative cross-sectional study	Total: N = 185 HIV positive mothers of infants less than one year old: *n* = 40 Mothers of undisclosed HIV status with an infant below the age of one: *n* = 35 Pregnant women: *n* = 25 Grandmothers of grandchildren less than one year: *n* = 26 Fathers of infants less than one year: *n* = 26 Health providers: *n* = 19 Traditional birth: *n* = 7	Semi-structured interviews Focus group discussions	The mothers did not fully understand the goals of the study, raising concern to the informed consent process Randomization as a study technique does not make sense to the women participating in the study Women participating in studies often feel compelled to share study interventions, such as medications and food supplements, with partners and families	Current study nested within The Breastfeeding, Antiretroviral and Nutrition (BAN) Study	Balancing risks Adequate informed consent	60%
Krubiner et al. [5]	United States South Africa Botswana Malawi	Qualitative cross-sectional study	Total N = 62 HIV investigators and clinicians	Focus group discussions Semi-structured interviews	Unclear guidance on the level of risk and benefit of the research for the woman and fetus, as determined by the ethical review boards. This uncertainty may cause investigators to exclude this population to enable other aspects of the study to progress with great efficiency Researchers are unsure who to include in the enrolment process because of the complex relationship of mother, fetus, and father The consequences for errors in research with pregnant women are grave (e.g. authors cite thalidomide study), which dis-incentivizes research with this population The financial costs for including pregnant women are much higher compared to research with other populations, due to the need for extended follow up	Current study nested within Pregnancy and HIV/AIDS: Seeking Equitable Study (PHASES)	Balancing Risks Consideration of paternal involvement Access to research and treatment	32%
Sullivan et al. [14]	United States Malawi	Qualitative cross-sectional study	Total: N = 140 women with HIV or at high risk for HIV United States: *n* = 70 Malawi: *n* = 70	Semi-structured interviews	Results were primary focused on reasons in promoting/opposing a paternal consent requirement for HIV research involving pregnant women The rights of the father, protection of the fetus if something to were happen to the mother, and paternalistic gender dynamics were cited by women as reasons in favor of paternal consent requirement Maternal rights, protecting interests of fetus, and controlling partners were cited by women as reasons to oppose paternal consent requirements	Current study nested within Pregnancy and HIV/AIDS: Seeking Equitable Study (PHASES)	Balancing risks Consideration of paternal involvement	86%

*BAN* Breastfeeding, Antiretroviral and Nutrition Study, *PHASES* Pregnancy and HIV/AIDS: Seeking Equitable Study, *STROBE* The Strengthening the Reporting of Observational Studies in Epidemiology

### Background summary of articles

Each of the three articles selected for review were sub-studies within two larger studies: the BAN study [13]; and PHASES [5, 14].

Formative research for the BAN study in Lilongwe, Malawi sought to understand attitudes and concerns of the local community on research participation, infant feeding practices, and maternal education [13]. PWLHIV and pregnant women with undisclosed HIV status were among those who were recruited in qualitative work to elicit these perspectives, although only a subset of results focused on their perspectives on their research participation [13]. During a three-month period, semi-structured interviews, focus group discussions, and home visits were performed to elicit key perspectives on specific ethical considerations in the conduct of the BAN study [13].

Two qualitative studies occurred as sub-studies within the PHASES project. One study sought to determine perceived barriers and constraints for HIV investigators and clinicians in performing HIV research with pregnant women, and included interviews with professionals from the United States, South Africa, Botswana, and Malawi [5]. The second study focused primarily on women’s views regarding a paternal consent requirement for pregnant women’s participation in research [14]. Pregnant and recently pregnant women were recruited from both the United States and Malawi, and all were living with HIV or at high risk for HIV acquisition [14].

### Ethical considerations for research involving pregnant women living with HIV

**Fig. 2:**
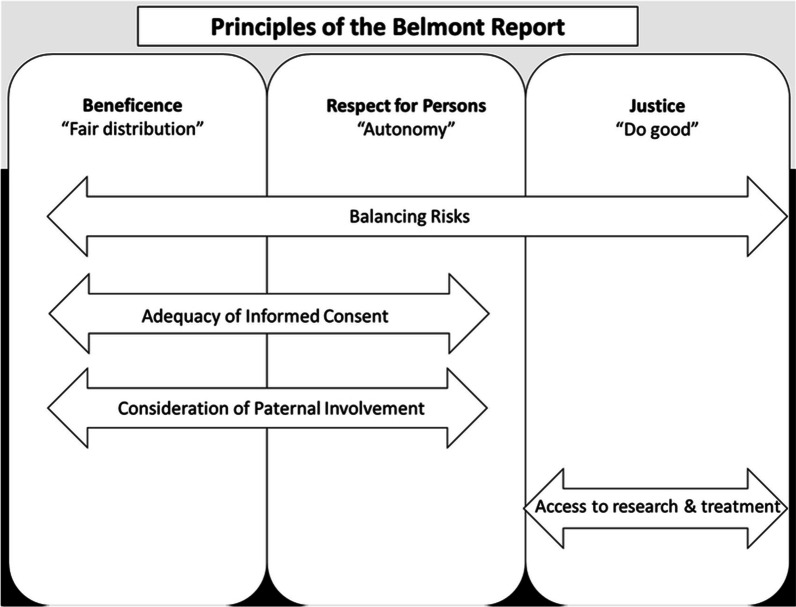
Thematic synthesis using Belmont Report

Analyzing the three studies through the lens of the Belmont Report, four main ethical themes emerged when conducting research with PWLHIV and were mapped to each of the core ethical principles: Beneficence, Respect for Persons, and Justice (Fig. 2). These themes included: adequacy of informed consent; consideration of paternal involvement; balancing risks; and access to research and treatment.

#### Adequacy of informed consent

The BAN study evaluated the safety and efficacy of antiretroviral and nutritional interventions to reduce HIV transmission during breastfeeding [13]. Within this study, participants had difficulty achieving appropriate levels of understanding regarding study activities during the informed consent process [13]. The researchers involved attributed this to the limited educational background of participants [13]. While all women agreed to participate, many were unable to differentiate between activities related to established clinical practice and activities related to the investigational study [13]. Nearly all women assumed the physicians involved in research would only provide them with medicine already proven safe and expected their child would have a therapeutic benefit from the study medicines [13]. Similarly, most participants had a limited understanding of the randomization procedure within the study, with some attributing differences in study conditions to participant need, rather than chance [13].

#### Consideration of paternal involvement

To researchers and clinicians, obtaining informed consent for PWLHIV is complicated by uncertainty surrounding whether the biological father should have a role [5]. Some participants raised concerns on whether it was ethically appropriate or necessary to require consent, but a common opinion on this issue was not reported [5]. In the study eliciting perspectives of pregnant or recently pregnant women, the majority of participants supported paternal consent regulation for research with the potential to benefit an unborn child (52% of participants in United States, 66% in Malawi), citing a shared sense of responsibility with the baby’s father before participating in research [14]. These women noted that by allowing the father to serve as an additional advocate for the fetus and have full knowledge of exposures to the fetus, it may help minimize research risks. [14]. Conversely, others argued that it was the woman who was carrying the pregnancy, so it should be her decision alone about whether to participate regardless of who would primarily benefit [14]. Further risk considerations are discussed below.

#### Balancing risks

Two articles highlighted the challenges of evaluating and balancing the potential risks of research for the fetus and mother [5, 14]. The physiology within the fetal-mother relationship is an area of concern during research, because what may benefit one party (e.g. a new therapy) might potentially risk harm to the other due to the unique physiologies between a pregnant mother and her fetus [5]. Furthermore, researchers are often discouraged from including pregnant women in intervention studies due to the limited existing data on physiological and pharmacokinetic effects to the fetus, stifling the development of evidence-based therapies to improve care and outcomes [5].

Beyond the challenge of balancing intervention-related risks between PWLHIV and their unborn children, PWLHIV are faced with additional external risks related to their participation in research. Some research may result in disclosure of HIV status by PWLHIV to their partners. Within one study, some women noted that men had left their wives who participated in studies of prevention of mother-to-child transmission after learning of the mother’s HIV status [14]. This unintended disclosure of HIV status would have devastating economic and social impacts on a family that would outweigh any potential benefit from participating in the research. Even without HIV disclosure of status, some women in Malawi noted that if a husband was unaware of a wife’s research participation, he may grow suspicious of her absences at home, break up the marriage, or reject the baby. They preferred instead to have the father involved and required to also provide consent [14]. Some expressed concern that partners who were controlling or violent would be able to prevent the mother’s autonomous decisions regarding her own healthcare and participation in research [14]. Within the same study, others expressed that mothers generally act in the baby’s best interest, and if the parents disagree, then the child would lose the potential benefit of research [14]. The perceived benefits of research included the availability of medicines, as well as a higher level of clinical care and monitoring [13, 14].

PWLHIV face the additional risk of exploitation when participating in research, impacting aspects of autonomy and justice. The BAN study describes the influence that culture has on asset allocation within households and communities [13]. Thirty percent of women claimed that if given nutritional supplements or antiretroviral medications within a research context, they would share them with their family, as they believed whatever one has, one must share [13]. This is especially the case when other children or family members are malnourished. Researchers within this study discussed the implications of this sharing and chose to label the supplement for study participants as, “Nutrition for Breastfeeding Mothers,” while participants were also provided with rice for their family [13]. The pressure for mothers to give up medications or nutritional supplements may not be perceived as exploitations within cultures that prioritize sharing resources. However, foreign researchers should be aware of this possibility and ensure that research benefits do not cause undue inducement for study participants.

#### Access to research and treatment

One article explored the topic of justice, by providing investigators’ perspectives on PWLHIV and their access to research and evidence-based treatments [5]. While U.S. regulations allow participation of pregnant women in research, investigators’ anecdotal experiences have led them to believe that ethical oversight officials do not want pregnant women enrolled [5]. Because of concerns that ethical boards may delay or refuse to approve clinical trials involving pregnant participants, some investigators reported being discouraged from including pregnant women in their research. Other disincentives include financial and analytical ones, as some investigators feel that funding agencies are hesitant to support pregnancy-related research due to the additional follow-up and ancillary care that may be required to fund in resource-limited settings [5]. With the additional logistical challenges and difficulty in analyzing smaller subsets of data, investigators often exclude this population despite the awareness of benefits for their inclusion in research [5].

## Discussion

This systematic review identified empiric studies evaluating ethical considerations in research with PWLHIV and their young children. Only three relevant publications were found meeting inclusion/exclusion criteria, reflecting a dearth of literature in this area. While we allowed for inclusion of studies involving young children born to mothers with HIV, none of the three articles found focused primarily on them. While a strength of this review is that it combined study data including international research investigators, community leaders, male partners and PWLHIV, only two studies collected empiric data from PWLHIV regarding their experiences participating in research [13, 14]. Four themes emerged: balancing the risks of research for the mother and fetus, adequacy of informed consent, consideration of paternal involvement, and access to research and treatment. Researchers and funding agencies should be aware of these considerations and appreciate the value of and critical need for formative research to ensure clinical trials involving PWLHIV promote ethical, well-informed research participation and, ultimately, improve care outcomes.

Balancing risk related to research participation is a key tenet of traditional institutional review boards, as it spans across the three core principles of the Belmont Report—Respect for Persons, Beneficence, and Justice. For HIV-related research, the level of risk and potential for benefit can be particularly impactful for women and their children. Research involving antiretroviral therapies or adjunct interventions have great potential to benefit mothers, and ultimately their children if vertical transmission of HIV is prevented or other positive effects observed [15]. The introduction of dolutegravir is an example of the importance of including pregnant women in research. This medicine was rolled out in many countries in 2018, after clinical trials demonstrated a reduced side effect profile and increased potency compared to standard therapy [16]. Pregnant women, however, were excluded from participating in these trials. Given the incomplete data collected, practitioners feared prescribing dolutegravir after a safety signal was identified among a surveillance cohort in Botswana, indicating dolutegravir exposure may be associated with an increased rate of neural tube defects [17]. This led to many countries altering their guidelines to advise against administration of dolutegravir for women of child-bearing age. As the surveillance cohort in Botswana increased in numbers, the initial signal related to dolutegravir decreased and was no longer statistically significance [18, 19]. The World Health Organization currently recommends dolutegravir as the preferred treatment for all populations, including pregnant and women of childbearing age [20]. The dolutegravir experience taught members of the HIV community that exclusion does not equate to protection, and that in actuality, exclusion sometimes does more harm than good [21]. Practitioners caring for PWLHIV need pregnant-specific data to ensure the adequate safety of new therapeutics. Since the dolutegravir debate lasted over a year, PWLHIV could not access it during that time. Furthermore, many countries continued to delay changes in their policy for fear of harming the fetus, preventing women from receiving a clinical superior medicine to control a life-threatening disease [21, 22]. The dolutegravir dilemma raised awareness that a component of ethical research is timely data collection, using a systematic approach to address questions of teratogenicity, safety, pharmacokinetics, dosing, and efficacy for PWLHIV [23].

The complexities of balancing risk are highlighted in relation to another theme which emerged in this review: consideration of paternal involvement. Within one study, pregnant or recently pregnant participants were asked their perspectives regarding a paternal consent requirement for a hypothetical research study that solely benefited the fetus, which mirrors the U.S. regulation 45 CFR part 46.204 requiring paternal consent [24]. While no clear consensus was achieved regarding this issue, there were concerns that controlling or violent partners may limit a mother’s autonomous decisions regarding her own healthcare and participation in research, thus limiting her own human rights [14]. Such cases have previously been described within HIV research. Because of the stigma linked to HIV in many communities, women avoid testing if they fear HIV-stigma and violence from their partners [25]. Furthermore, this stigma also discourages them from disclosing their status to their partners [26, 27], resulting in avoiding involvement in HIV-related studies [28]. This fear of rejection or violence from a partner puts pregnant women in a place of vulnerability, which should be considered when designing research studies involving this population.

Adequacy of informed consent is a critical component in all ethical research; however, none of the articles provided clear guidance on how to specifically ensure this for PWLHIV. In other fields of research, interventions have been developed to improve understanding for prospective research participants, such as using multimedia interventions, test/feedback, extended study discussion, and enhanced consent forms, most with only limited effectiveness [29]. Investigators working in resource-limited settings face additional challenges, as potential study populations may have lower exposure to research concepts than populations in higher resourced settings [30]. Therefore, investigators should be encouraged to incorporate formative, community-engaged research at the start of new study trials to understand the contexts for these research studies and subsequently develop methods optimizing participant understanding during the informed consent process. Two studies included within this review did formative work and reported benefits to improving the research designs [13, 14]. While this may lengthen the time to initiate studies, such work is ultimately necessary to provide critical insights for performing high-quality and ethical research, which will serve future investigations as well.

With the increased emphasis on community-engaged research and research partnerships with local organizations, potential participants may view research interventions as an extension of the existing community resources or preferable to the standard care, especially when no care would otherwise be provided [31, 32]. The desire to access care was a prominent theme that emerged from the studies in this review, which has ethical implications when working with potentially vulnerable populations in resource-limited settings. In a study in Malawi, PWLHIV reported participating in research because of a desire for access to enhanced health care, such as supplemental laboratory testing and a better chance of protecting their child from HIV [33]. In light of these viewpoints, it is critical to ensure the informed consent process is clear in conveying risks of participation and fair distribution of those risks, as well as equipoise among investigational interventions. In doing this, researchers support study participants’ rights to autonomy and decision-making capacity.

Additional ethical considerations are needed when studying interventions in resource-limited settings. As noted in our findings, women enrolled in research studies may be forced or feel compelled to share study incentives, reimbursements, or treatments with family or community members, putting them at risk for exploitation. The loss of beneficial services after study completion is a related consideration, which is often outlined in ethical guidelines but not described among the studies included in this review. The CIOMS guidelines is one such guideline that emphasizes the importance of considering the sustainability of research in resource-limited settings [3]. Understanding the potential harms or benefits that are perceived with the short-term availability of research interventions is an area that requires additional investigation, including through the perspectives of potential study participants.

Without community perspectives, researchers may have a limited ability to recognize and consider nuanced ethical research considerations for PWLHIV due to their differing life and cultural experiences. Women living in sub-Saharan Africa bear a disproportionate burden of HIV [34]. Meanwhile, the majority of researchers performing large HIV clinical trials come from high-income settings, such as North America or Europe, where cultural context may lead to differences in consideration of certain ethical principles compared to what may be prioritized within low-and middle-income settings. For example, in the United States, many ethical guidelines and frameworks place significant weight on principles such as autonomy and the rights of an individual. In regions with generalized HIV epidemics, however, ethical considerations involving community consent may be important [34–36]. To develop ethical guidelines for research with PWLHIV, the cultural, social, and economic contexts of the research setting must be taken into account [2]. Therefore, it is necessary for international researchers to have equitable partnerships with local investigators and ethical boards to determine appropriate ethical approaches in the research setting. When local concerns are not considered, such as who should be involved in the decision-making processes, the research may create communal tension [34]. This again supports the use of formative research prior to study initiation due to the influence that cross-cultural contexts may have on considerations for study approaches and successful completion of study activities.

It is also important to consider a population’s degree of vulnerability based upon context specifics [37]. There is no universal definition of vulnerability, and it is rarely defined in most ethical frameworks [35]. Past reviews of the literature focused on vulnerability experienced by pregnant women have found that this term is dependent on context, with determination that pregnant women are inherently vulnerable due to the lack of scientific understanding surrounding their clinical care, as well as the lack of research involving them [37]. Furthermore, within this review, ethical concerns that emerged, such as balancing risk and consideration of paternal consent, are unique challenges faced by PWLHIV. Ethical guidelines involving pregnant women must acknowledge that this group in particular consists of a diverse population with different needs. This adds to the evidence to support the importance of partnering with local investigators and engaging with the community and potential study population to incorporate the overarching ethical principles into best practices in achieving research goals, while respecting the study participants and minimizing risks and the possibility of exploitation.

Exploitation can manifest itself in the area of undue inducement during research studies, as PWLHIV often lack financial stability, gender equality, and health care [38]. Furthermore, women are often targeted by community members when participating in studies due to the community’s belief that there are numerous resources attached to research [32]. Therefore, compensation must be thoughtfully considered, as not to put the women at potential risk. When lacking financial stability, gender equality, and healthcare access, women may also be more willing to risk injury to themselves or the fetus as there are limited opportunities to get prevention or care for HIV [39]. In a South African study, a woman falsely claimed to be pregnant in order to acquire access to the study drug, a tenofovir gel that was proven to reduce HIV acquisition among women [39]. Further investigation uncovered that her complex social situation and lack of financial resources provided her with limited options to prevent HIV infection from her partner, ultimately leading to her actions [39]. This case study highlights the difference in perception of the risk–benefit ratio and shows that in settings without access to appropriate medications for prevention or treatment of HIV, simply including antiretroviral therapy as an intervention can be cause for undue inducement [39]. To mitigate undue inducement in under-resourced areas, the CIOMS recommends under guideline 13 that all studies be approved by a local research ethics committee [40]. However, given that the subjectivity of ethical considerations depends upon the context, formative research becomes even more imperative.

The informed consent process was identified as a particular vulnerable aspect of PWLHIV’s research participation, as the four themes that emerged from this review were related to various aspects within the informed consent process. Research involving PWLHIV should incorporate proven methods to enhance potential participant’s understanding of the research activities, such as the test feedback approach and person-to-person discussion [41]. The person-to-person discussion allows potential participants to gain deep understanding through a one-on-one conversation, in which they are told about the study before consenting [41]. The test feedback approach gauges participant’s understanding by giving them questions to answer after hearing a description of the study. An example of the test feedback approach that could apply to PWLHIV would be asking each woman (1) What do you perceive as the potential risks/benefits from participating in this research? (2) Do you have any alternatives to caring for your health other than participating in this study? (3) Would you consider yourself to be invited to do this research or forced into doing this research? (4) What procedures will you be participating in if you did enroll in this research? (5) Even after enrolling, are you able to leave the study if you so desire? If the participant fails to answer any of the questions, the recruiter would need to re-address the topic prior to allowing participant’s consent [41]. Alternative strategies for enhancing the informed consent process may be developed with local communities. Regardless of the specific strategy chosen, researchers have a duty to ensure appropriate informed consent prior to study participation.

### Limitations

This systematic review was limited by the very low number of articles meeting study inclusion criteria, given the lack of studies regarding ethical considerations of PWLHIV participation in research. Furthermore, we were unable to find studies regarding studies related to research with infants and young children born to HIV-infected mothers. No studies were included that explored issues related to disclosure of HIV status to partners or family members as a result of research participation, among other hypothetical considerations when participating in research.

### Future implications

When including PWLHIV and their young children, researchers need to invest time in conducting formative studies alongside local investigators. Their capacity to understand the unique study population, who often live within a cultural context that differs from that of the researchers themselves, will be enhanced. Furthermore, it will facilitate the optimization of study protocols and increase the likelihood of a feasible intervention. Moreover, by assessing the participants’ understanding of the study prior to consent, which was cited as the most prominent ethical concern, and exploring potential study burdens, researchers can minimize ethical challenges, which will advance research, translating to increased clinical evidence when treating PWLHIV and their young children. Person-to-person discussion and the test-feedback approach are two proven strategies for accomplishing this matter [41]. Also, funding agencies and ethical review boards should be aware of these considerations, appreciating the value of and critical need to perform formative studies.

## Conclusions

Evidence is limited regarding ethical considerations for research with PWLHIV. Researchers should be aware of possible misinterpretations of investigational interventions as proven therapies by potential study participants. To ensure voluntary informed consent, researchers must assess participant’s understanding of key study information through approaches such as person-to-person discussion and test feedback approach [41]. Researchers must also be aware of the community influences impacting an individual’s participation in the study, including issues related to consent and how study treatments or reimbursements may be redistributed within families or the community. To minimize undue inducement, consultation with key community stakeholders, leaders, and the potential participants will aid researcher’s understanding of the social, economic, political, and cultural context in which their study is operating. While no ethical guidelines can be comprehensive, this review highlights multiple ethical issues which have arisen in the course of research with PWLHIV. It also highlights the need for formative research targeting study populations to ensure thoughtful study designs that consider the needs and values of PWLHIV and their communities.

## Data Availability

The journal articles supporting the conclusions of this review are cited and are available on their respective journal’s websites. Copyright laws prevents us from making these articles freely available.

## Appendix 1: Sample Search Strategy: Ovid MEDLINE

**Table:**

OVID Search (Run on 11/26/2019)	
1. exp Ethics/ (139596)	
2. ethics.fs. (65253)	
3. ethics.mp. (152536)	
4. exp Disclosure/sn [Statistics & Numerical Data] (272)	
5. exp Confidentiality/ (50261)	
6. confidentiality.mp. (26504)	
7. exp Informed Consent/ (39227)	
8. informed consent.mp. (53834)	
9. maleficence.mp. (353)	
10. beneficence.mp. (4057)	
11. autonomy.mp. (40738)	
12. social justice.mp. (12240)	
13. moral conflict.mp. (108)	
14. 1 or 2 or 3 or 4 or 5 or 6 or 7 or 8 or 9 or 10 or 11 or 12 or 13 (279793)	
15. exp HIV/ (93156)	
16. exp HIV Infections/ (265887)	
17. exp Anti- HIV Agents/(63164)	
18. Vulnerable Populations.mp. or Vulnerable Populations/ (11649)	
19. exp Developing Countries/ (71120)	
20. developing countr*.mp. (108789)	
21. HIV.mp. (305424)	
22. AIDS.mp. (173524)	
23. PrEP.mp. (2944)	
24. Pre-Exposure Prophylaxis/ (1187)	
25. 15 or 16 or 17 or 18 or 19 or 20 or 21 or 22 or 23 or 24 (509241)	
26. pregnant women.mp. or exp Pregnant Women/ (76128)	
27. exp Pregnancy/ (846479)	
28. pregnan*.mp. (901759)	
29. exp Child, Orphaned/ (641)	
30. exp Child, Abandoned/ (510)	
31. orphan*.mp. (17102)	
32. exp adolescent/ or exp child/ or exp infant/ (3344555)	
33. (teen or child* or toddler* or baby or infant or adolescen* or kid).mp. (3577840)	
34. 26 or 27 or 28 or 29 or 30 or 31 or 32 or 33 (4197141)	
35. 14 and 25 and 34 (4576)	
36. limit 35 to (english language and humans) (4137)	
37. limit 36 to yr="1985–2018" (4082)	
38. [from 37 keep 4001–4180] (0)	
39. exp clinical study/ (867233)	
40. clinical trial*.mp. (897620)	
41. exp Clinical Trials as Topic/ (319339)	
42. exp Research Subjects/ (18151)	
43. research design.mp. or exp Research Design/ (431832)	
44. exp Interview, Psychological/ (14655)	
45. interview*.mp. (306123)	
46. exp Data Collection/ (1966609)	
47. exp empirical research/ (46239)	
48. empirical research.mp. (7120)	
49. qualitative research.mp. (48898)	
50. 39 or 40 or 41 or 42 or 43 or 44 or 45 or 46 or 47 or 48 or 49 (3377768)	
51. 37 and 50 (2330) limited to research	

## Appendix 2: Individual STROBE Quality Assessment

*Modified scoring during STROBE criteria*:

Articles were rated using a ‘+’, ‘−’, or ‘+/−’ system to denote whether the article included the necessary information. The ‘+’ meant the article included that information or section. The ‘−’ meant the article lacked that information or section. The ‘+/−’ meant the article included some of the information, but it did not address everything. Furthermore, not all components of the STROBE guidelines fit the articles being assessed. Therefore, those questions were denoted with ‘N/A’ and left out of the calculations. To calculate the quality percentage of each article, the ‘+’ was associated with 100%. The ‘−’ was associated with 0%. The ‘+/−’ was associated with 50%. There was a quality percentage done for each of the five sections in each article. There was one question from the title section, two questions in the introduction section, nine questions in the methods section, and five questions in both the results and discussion sections. Based on quantity of questions found in that portion of the STROBE guidelines determined how much that section contributed to the overall quality.

*Corneli’s Article*

STROBE Statement—Checklist of items that should be included in reports of *cross-sectional studies*

**Table:**

	Item No	Recommendation	Page No
**Title and abstract**	1	(*a*) Indicate the study’s design with a commonly used term in the title or the abstract	−
		(*b*) Provide in the abstract an informative and balanced summary of what was done and what was found	+
**Introduction**			
Background/rationale	2	Explain the scientific background and rationale for the investigation being reported	+
Objectives	3	State specific objectives, including any prespecified hypotheses	+
**Methods**			
Study design	4	Present key elements of study design early in the paper	+
Setting	5	Describe the setting, locations, and relevant dates, including periods of recruitment, exposure, follow-up, and data collection	+
Participants	6	(*a*) Give the eligibility criteria, and the sources and methods of selection of participants	−
Variables	7	Clearly define all outcomes, exposures, predictors, potential confounders, and effect modifiers. Give diagnostic criteria, if applicable	+/−
Data sources/ measurement	8*	For each variable of interest, give sources of data and details of methods of assessment (measurement). Describe comparability of assessment methods if there is more than one group	*+*
Bias	9	Describe any efforts to address potential sources of bias	+/−
Study size	10	Explain how the study size was arrived at	−
Quantitative variables	11	Explain how quantitative variables were handled in the analyses. If applicable, describe which groupings were chosen and why	n/a
Statistical methods	12	(*a*) Describe all statistical methods, including those used to control for confounding	+
		(*b*) Describe any methods used to examine subgroups and interactions	n/a
		(*c*) Explain how missing data were addressed	n/a
		(*d*) If applicable, describe analytical methods taking account of sampling strategy	n/a
		(*e*) Describe any sensitivity analyses	n/a
**Results**			
Participants	13*	(a) Report numbers of individuals at each stage of study—eg numbers potentially eligible, examined for eligibility, confirmed eligible, included in the study, completing follow-up, and analysed	+
		(b) Give reasons for non-participation at each stage	n/a
		(c) Consider use of a flow diagram	n/a
Descriptive data	14*	(a) Give characteristics of study participants (eg demographic, clinical, social) and information on exposures and potential confounders	+/−
		(b) Indicate number of participants with missing data for each variable of interest	n/a
Outcome data	15*	Report numbers of outcome events or summary measures	n/a
Main results	16	(*a*) Give unadjusted estimates and, if applicable, confounder-adjusted estimates and their precision (e.g., 95% confidence interval). Make clear which confounders were adjusted for and why they were included	n/a
		(*b*) Report category boundaries when continuous variables were categorized	n/a
		(*c*) If relevant, consider translating estimates of relative risk into absolute risk for a meaningful time period	n/a
Other analyses	17	Report other analyses done—eg analyses of subgroups and interactions, and sensitivity analyses	n/a
**Discussion**			
Key results	18	Summarise key results with reference to study objectives	+
Limitations	19	Discuss limitations of the study, taking into account sources of potential bias or imprecision. Discuss both direction and magnitude of any potential bias	−
Interpretation	20	Give a cautious overall interpretation of results considering objectives, limitations, multiplicity of analyses, results from similar studies, and other relevant evidence	−
Generalisability	21	Discuss the generalisability (external validity) of the study results	−
**Other information**			
Funding	22	Give the source of funding and the role of the funders for the present study and, if applicable, for the original study on which the present article is based	+

*Give information separately for exposed and unexposed groups

*Krubiner’s Article*

**Table:**

	Item No	Recommendation	Page No
**Title and abstract**	1	(*a*) Indicate the study’s design with a commonly used term in the title or the abstract	−
		(*b*) Provide in the abstract an informative and balanced summary of what was done and what was found	+
**Introduction**			
Background/rationale	2	Explain the scientific background and rationale for the investigation being reported	+
Objectives	3	State specific objectives, including any prespecified hypotheses	−
**Methods**			
Study design	4	Present key elements of study design early in the paper	+
Setting	5	Describe the setting, locations, and relevant dates, including periods of recruitment, exposure, follow-up, and data collection	−
Participants	6	(*a*) Give the eligibility criteria, and the sources and methods of selection of participants	−
Variables	7	Clearly define all outcomes, exposures, predictors, potential confounders, and effect modifiers. Give diagnostic criteria, if applicable	n/a
Data sources/ measurement	8*	For each variable of interest, give sources of data and details of methods of assessment (measurement). Describe comparability of assessment methods if there is more than one group	−
Bias	9	Describe any efforts to address potential sources of bias	−
Study size	10	Explain how the study size was arrived at	−
Quantitative variables	11	Explain how quantitative variables were handled in the analyses. If applicable, describe which groupings were chosen and why	n/a
Statistical methods	12	(*a*) Describe all statistical methods, including those used to control for confounding	−
		(*b*) Describe any methods used to examine subgroups and interactions	n/a
		(*c*) Explain how missing data were addressed	n/a
		(*d*) If applicable, describe analytical methods taking account of sampling strategy	n/a
		(*e*) Describe any sensitivity analyses	n/a
**Results**			
Participants	13*	(a) Report numbers of individuals at each stage of study—eg numbers potentially eligible, examined for eligibility, confirmed eligible, included in the study, completing follow-up, and analysed	+
		(b) Give reasons for non-participation at each stage	n/a
		(c) Consider use of a flow diagram	n/a
Descriptive data	14*	(a) Give characteristics of study participants (eg demographic, clinical, social) and information on exposures and potential confounders	−
		(b) Indicate number of participants with missing data for each variable of interest	n/a
Outcome data	15*	Report numbers of outcome events or summary measures	n/a
Main results	16	(*a*) Give unadjusted estimates and, if applicable, confounder-adjusted estimates and their precision (e.g., 95% confidence interval). Make clear which confounders were adjusted for and why they were included	n/a
		(*b*) Report category boundaries when continuous variables were categorized	n/a
		(*c*) If relevant, consider translating estimates of relative risk into absolute risk for a meaningful time period	n/a
Other analyses	17	Report other analyses done—eg analyses of subgroups and interactions, and sensitivity analyses	n/a
**Discussion**			
Key results	18	Summarise key results with reference to study objectives	+
Limitations	19	Discuss limitations of the study, taking into account sources of potential bias or imprecision. Discuss both direction and magnitude of any potential bias	−
Interpretation	20	Give a cautious overall interpretation of results considering objectives, limitations, multiplicity of analyses, results from similar studies, and other relevant evidence	−
Generalisability	21	Discuss the generalisability (external validity) of the study results	+
**Other information**			
Funding	22	Give the source of funding and the role of the funders for the present study and, if applicable, for the original study on which the present article is based	−

*Give information separately for exposed and unexposed groups

*Nama’s Article*

**Table:**

	Item No	Recommendation	Page No
**Title and abstract**	1	(*a*) Indicate the study’s design with a commonly used term in the title or the abstract	−
		(*b*) Provide in the abstract an informative and balanced summary of what was done and what was found	+/−
**Introduction**			
Background/rationale	2	Explain the scientific background and rationale for the investigation being reported	+
Objectives	3	State specific objectives, including any prespecified hypotheses	+/−
**Methods**			
Study design	4	Present key elements of study design early in the paper	−
Setting	5	Describe the setting, locations, and relevant dates, including periods of recruitment, exposure, follow-up, and data collection	−
Participants	6	(*a*) Give the eligibility criteria, and the sources and methods of selection of participants	−
Variables	7	Clearly define all outcomes, exposures, predictors, potential confounders, and effect modifiers. Give diagnostic criteria, if applicable	n/a
Data sources/ measurement	8*	For each variable of interest, give sources of data and details of methods of assessment (measurement). Describe comparability of assessment methods if there is more than one group	−
Bias	9	Describe any efforts to address potential sources of bias	−
Study size	10	Explain how the study size was arrived at	−
Quantitative variables	11	Explain how quantitative variables were handled in the analyses. If applicable, describe which groupings were chosen and why	n/a
Statistical methods	12	(*a*) Describe all statistical methods, including those used to control for confounding	−
		(*b*) Describe any methods used to examine subgroups and interactions	n/a
		(*c*) Explain how missing data were addressed	n/a
		(*d*) If applicable, describe analytical methods taking account of sampling strategy	n/a
		(*e*) Describe any sensitivity analyses	n/a
**Results**			
Participants	13*	(a) Report numbers of individuals at each stage of study—e.g. numbers potentially eligible, examined for eligibility, confirmed eligible, included in the study, completing follow-up, and analysed	+
		(b) Give reasons for non-participation at each stage	n/a
		(c) Consider use of a flow diagram	n/a
Descriptive data	14*	(a) Give characteristics of study participants (e.g. demographic, clinical, social) and information on exposures and potential confounders	−
		(b) Indicate number of participants with missing data for each variable of interest	n/a
Outcome data	15*	Report numbers of outcome events or summary measures	n/a
Main results	16	(*a*) Give unadjusted estimates and, if applicable, confounder-adjusted estimates and their precision (e.g., 95% confidence interval). Make clear which confounders were adjusted for and why they were included	n/a
		(*b*) Report category boundaries when continuous variables were categorized	n/a
		(*c*) If relevant, consider translating estimates of relative risk into absolute risk for a meaningful time period	n/a
Other analyses	17	Report other analyses done—e.g. analyses of subgroups and interactions, and sensitivity analyses	n/a
**Discussion**			
Key results	18	Summarise key results with reference to study objectives	+
Limitations	19	Discuss limitations of the study, taking into account sources of potential bias or imprecision. Discuss both direction and magnitude of any potential bias	−
Interpretation	20	Give a cautious overall interpretation of results considering objectives, limitations, multiplicity of analyses, results from similar studies, and other relevant evidence	−
Generalisability	21	Discuss the generalisability (external validity) of the study results	−
**Other information**			
Funding	22	Give the source of funding and the role of the funders for the present study and, if applicable, for the original study on which the present article is based	+

Give information separately for exposed and unexposed groups

An Explanation and Elaboration article discusses each checklist item and gives methodological background and published examples of transparent reporting. The STROBE checklist is best used in conjunction with this article (freely available on the Web sites of PLoS Medicine at http://www.plosmedicine.org/, Annals of Internal Medicine at http://www.annals.org/, and Epidemiology at http://www.epidem.com/). Information on the STROBE Initiative is available at www.strobe-statement.org.

## Abbreviations

HIV: Human immunodeficiency virus
PWLHIV: Pregnant women living with HIV
CIOMS: Council for International Organizations of Medical Sciences
PRIMSA: Preferred Reporting Items for Systematic Reviews and Meta-Analyses
CINAHL: Cumulative Index to Nursing and Allied Health Literature
BAN: Breastfeeding, Antiretroviral, and Nutrition
PHASES: Pregnancy and HIV/AIDS: Seeking Equitable Study
